# Autoantibodies to Posttranslational Modifications in Rheumatoid Arthritis

**DOI:** 10.1155/2014/492873

**Published:** 2014-03-23

**Authors:** Agata N. Burska, Laura Hunt, Marjorie Boissinot, Rocky Strollo, Brent J. Ryan, Ed Vital, Ahuva Nissim, Paul G. Winyard, Paul Emery, Frederique Ponchel

**Affiliations:** ^1^Leeds Institute of Rheumatic and Musculoskeletal Disease, University of Leeds and NIHR Leeds Musculoskeletal Biomedical Research Unit, Leeds Trust Teaching Hospital, Leeds LS9 7TF, UK; ^2^Leeds Institute of Cancer and Pathology, University of Leeds, Leeds LS9 7TF, UK; ^3^Centre for Biochemical Pharmacology Research Unit, William Harvey Research Institute, Barts and London, Queen Mary's School of Medicine and Dentistry, University of London, Charterhouse Square, London EC1M 6BQ, UK; ^4^Department of Physiology, Anatomy and Genetics, University of Oxford, Le Gros Clark Building, Oxford OX1 3QX, UK; ^5^University of Exeter Medical School, St. Luke's Campus, Exeter, Devon EX1 2LU, UK

## Abstract

Autoantibodies have been associated with human pathologies for a long time, particularly with autoimmune diseases (AIDs). Rheumatoid factor (RF) is known since the late 1930s to be associated with rheumatoid arthritis (RA). The discovery of anticitrullinated protein antibodies in the last century has changed this and other posttranslational modifications (PTM) relevant to RA have since been described. Such PTM introduce neoepitopes in proteins that can generate novel autoantibody specificities. The recent recognition of these novel specificities in RA provides a unique opportunity to understand human B-cell development *in vivo*. In this paper, we will review the three of the main classes of PTMs already associated with RA: citrullination, carbamylation, and oxidation. With the advancement of research methodologies it should be expected that other autoantibodies against PTM proteins could be discovered in patients with autoimmune diseases. Many of such autoantibodies may provide significant biomarker potential.

## 1. Introduction

Specificity and memory are the hallmarks of the adaptive immune system. Immunological memory is well recognised but still not fully understood. It was first observed in antiquity, during the plague infection in Athens. It is also the basis of vaccination, which was first attempted in India over a thousand years ago when smallpox inoculation to healthy people resulted in a milder epidemic, while protection lasted many years, particularly in the absence of reexposure to the antigen. Molecular immunology has now unravelled the early steps towards the establishment of immunological memory; however, several areas remain unexplained particularly the mechanism of plasma cell development and maintenance. The recent recognition of the specificity of novel autoantibodies in rheumatoid arthritis (RA) provides a unique opportunity to understand human IgG and B-cell memory development* in vivo*.

The early phases of B-cell development are well established. Initially, naive B-cells are released in the circulation, where they meet with their antigens, becoming activated B-cells. At this stage several models have been proposed and IgG can develop through different routes, in a T-cell dependent or independent manner. In a (classic) linear model [1], maturation occurs in the presence of T-cells in a germinal centre-like reaction (GCR); B-cells switch from secreting IgM to secreting IgG and undergo affinity maturation. Some of these B-cells then develop into memory B-cells and others into long-lived plasma cells (LL-PC). These LL-PC then move to a bone marrow niche where they can survive for years. They are dependent on CXCL12 expression. In a variation of this model, activated B-cells go through a short-live plasma cell (SL-PC) stage before fully maturing into LL-PC [2]. In a third model both LL-PC and SL-PC secreting IgM and IgG were shown to develop directly from activated B-cells, independently of T-cell help. However this only occurs in the presence of antigen and alternative signals provided by innate immunity mechanisms such as direct TLR activation of B-cells [3]. It appears that these three models may actually coexist providing a first line of defense with rapid secretion of antibodies. However, further T-cell mediated maturation is necessary for a second line of defense involving long-term memory and LL-PC [4]. An inflamed environment such as the synovial membrane in RA (where CXCL12 is highly expressed in active disease [5]) is believed to provide an alternative niche for the survival of LL-PC.

Autoantibodies have been associated with human pathologies for a long time, particularly with autoimmune diseases (AIDs). Organ specific AIDs involve single or multiple autoantigens. In RA, autoantibodies have long been associated with the disease. Rheumatoid factor (RF), an autoantibody reacting against the Fc portion of IgG antibodies, was identified in the late 1930s. It was the most significant biomarker associated with RA until the discovery of anticitrullinated protein antibodies (ACPA). More recently, other posttranslational modifications (PTM) have been associated with the generation of specific autoantibodies that can be used as biomarkers [6–10]. While proteins are encoded by different sequences of amino acids, there are many ways to modify amino acids once introduced in protein sequences. Glycosylation, citrullination, methylation, acetylation, and ubiquitination are all types of physiological modifications. Other modifications can occur due to interaction with foreign substances (i.e., infections), environmental damage (such as UV exposure or chemical pollutants) leading to the formation of chemical adducts on the protein. Modifications including carbamylation, acetylation, ethylation, or methylation were sufficiently immunogenic to produce specific antibodies to these modified sequences of amino acids [11]. The analysis of autoantigen specific B-cell differentiation and maintenance, at the different stages of RA progression, provides a unique opportunity to understand disease and study immunological B-cell memory* in vivo *[12, 13].

Many AIDs are characterized by chronic inflammation, which may play a major role when inflammation-associated events such as chemical or enzyme-mediated modification of protein provide a source of neoepitopes that can be recognised by antibodies as non-self. In situations of stress such as inflammation, all types of physiological responses can be used in an abnormal manner. Citrullination is an enzymatic PTM which has an important role in the normal function of the immune system, epidermis differentiation insulation of neurons and the plasticity of the central nervous system [14]. Chlorination of protein occurs via the conversion of hydrogen peroxide to reactive chlorine species, such as HOCl, by granulocytes notably during inflammation. Other forms of oxidation result from the formation of reactive species of oxygen, nitrogen, and sulphur as a cellular response to various stimulations by growth factors or cytokines [15]. Oxidation products of sugars and unsaturated lipids can also react with proteins to cause chemical modifications. Nonenzymatic glycation is a naturally occurring phenomenon leading to development of PTM of proteins, nucleic acid, or lipids; it occurs in presence of high blood glucose but is also associated with aging and other inflammatory or degenerative diseases [16] such as RA [17], osteoarthritis [18], and Alzheimer's disease [19–21]. Carbamylation is a nonenzymatic, irreversible PTM. Carbamylation of proteins, lipids, peptides, and amino acids is widespread in health in mammals and is a natural physiological phenomenon. However excessive carbamylation will appear once proteins are exposed to high concentrations of isocyanate derived from the increased dissociation of urea and this alters the function of proteins [22].

Important evidence that perturbations in protein structures introduced by PTM are important in RA was brought by studies of collagen II (CII) for which PTM were shown to dramatically alter immunogenicity [6, 23–25] rendering some of them arthritogenic [26–30]. CII is the predominant cartilage collagen and a known autoantigen [23, 31, 32]. The human joint contains abundant CII and collagen-induced arthritis is the common experimental animal model of RA [33, 34]. Thus, antibodies to CII should be of highest relevance in RA [32]. Nevertheless, antinative CII antibodies occur only in 3–27% of patients with RA [29, 35–37] and, as such, it has been difficult to substantiate the role of autoimmunity to CII in the pathogenesis of RA. However today autoimmunity to PTM CII has been clearly demonstrated (cit-CII [24], ROS-CII [6, 7, 10, 38], although specific anticarbamylated CII remains to be demonstrated in human sera). These findings support the possibility that chemical modification of self-antigens, in RA in particular and in inflammation in general, may be the cause of formation of neoepitopes leading to autoimmunity [16, 39].

## 2. Anticitrullinated Protein Antibodies (ACPA) in RA

ACPA were originally described using different names such as anti-keratin (AKA), antiperinuclear factor (APF) antibodies, antifilaggrin antibodies (AFA), or anti-Sa [40]. ACPA have been associated with human pathology [41] as well as preclinical disease since the early 90s [42] later confirmed [43, 44]. The importance of these antibodies was then recognised several years later when their presence was identified as a specific event associated with RA [45–50]. Many reports were published; however, their relevance was reduced to a few publications where appropriate controls and procedures had been followed, particularly with respect to the ELISA assays used to detect ACPA [51, 52]. In the early years, the use of ELISA for individual reactivities (citrullinated filagrin or keratin) or “first generation” commercially available ELISA kits (CCP1, Immunoscan RA, Euro-diagnostica [53]) showed equal reactivity between RA (22%), healthy control sera (27%), and all kinds of arthritis and inflammatory diseases [49] although clear differences in titres were observed (sensitivities 45–64% but specificity over 90%). Later, “second-generation” ELISAs, showed higher specificity (*∼*98%) and sensitivity (40–76% depending on disease stage) [54]; however, more recent work also showed potential association of ACPA with psoriatic arthritis [55], periodontitis [56], and osteoarthritis [38]. The main difference between these tests resided in the antigens used to detect ACPA. The diagnostic value of ACPA were therefore established by demonstrating the importance of using appropriate citrullinated peptide [40, 51, 57]. The development of a highly sensitive noncommercial ELISA, based on protein targets identified as reactive with ACPA in synovial tissue (i.e., alpha and beta fibrinogen) was therefore explored [58]. Importantly, positivity of ACPA for one or both to these two citrullinated peptides covered all reactivity in RA sera [59].

### 2.1. Citrullination

**Figure 1:**
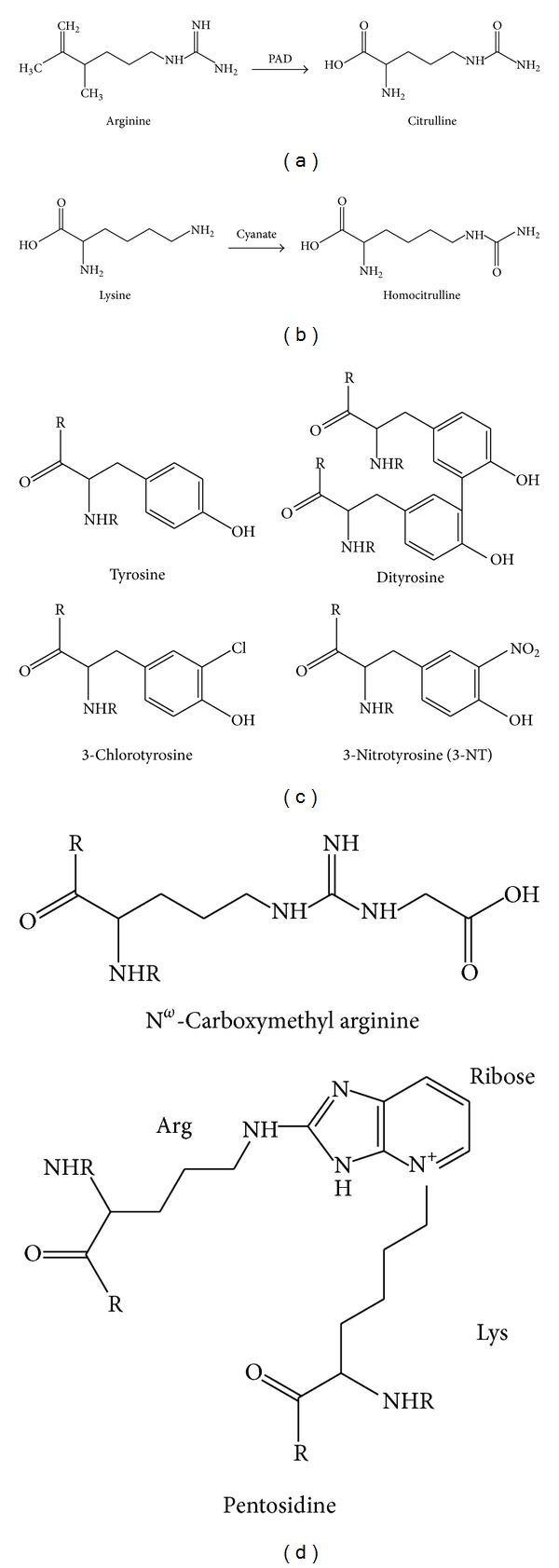
(a) Enzymatic generation of citrulline from arginine catalyzed by peptidylarginine deiminase (PAD); (b) non-enzymatic formation of homocitrulline by carbamylation of lysine by cyanate; (c) products of reactions between tyrosine with reactive oxygen species (forming dityrosine), reactive nitrogen species (forming 3-nitrotyrosine) and reactive chlorine species forming 3-chlorotyrosine; (d) examples of AGEs formed including carboxymethyl arginine and pentosidine (formed between an arginine and lysine residue).

ACPA represent a family of autoantibodies. However, only IgG-isotype of ACPA is specifically associated with RA. The antigen which triggers the immune reaction recognized by ACPA lies in the modification of protein (i.e., citrullination). In summary, after years of research, it was shown that this modification converts an arginine into a citrulline amino acid residue (citrullination) and is performed by an enzyme, peptidylarginine deiminase (PAD), thereby producing the immunogenic epitopes (Figure 1(a)) [60]. A consensus sequence, present in a wide range of proteins, is required for the modification of the arginine residue by the PAD. Metabolic stress related citrullination has also been proposed to play a role in multiple sclerosis [61, 62], Alzheimer's disease [20, 21], and cancer [63, 64]. The modifications introduced by PAD enzymes have important physiological roles, especially during differentiation, development, and apoptosis. PAD enzymes are expressed in a wide range of tissues (epidermis, sweat glands, hair follicles, ovary, and testis). In the synovium, only the enzymes PAD 2 and 4 are expressed; however, their expression is not specific for RA as they are also found in other forms of inflammatory and noninflammatory arthritis [65]. How both intracellular and extracellular proteins are citrullinated remains at the centre of many debates. PAD enzymes are necessary to catalyse protein deimination. PADs are not actively secreted in the intercellular space, although quite a few of their targets are extracellular proteins. Two immune-mediated membranolytic pathways (mediated by perforin and the membrane attack complex MAC), which are active in the RA joint and of importance in RA pathogenesis, have been proposed as possible ways by which PADs may be released in the joint microenvironment [66]. Several human citrullinated proteins have now been identified as target antigens of ACPA in RA (collagen, fibrinogen, vimentin, enolase, etc.) [40, 58, 59, 67, 68]. ACPA recognise citrullinated cross-reactive proteins but it is the presence of ACPA that is specific for RA rather than their protein antigens. The local context in which the proteins targeted for citrullination are expressed does not seem to have much importance; for example, filaggrin, which is an epithelial target of ACPA in RA, is not expressed in synovial tissue [58].

### 2.2. Clinical Relevance of ACPA

In RA patients, the presence of ACPA was associated with progressive and destructive disease outcomes [69–71], X-rays demonstrating the presence of erosions earlier and at a greater frequency in ACPA+ patients [72]. ACPA positivity was also associated with the presence of RF and shared epitope (SE) [50]. Combination analysis showed independent additive effects of these three factors for high radiological risk [35, 50, 69, 73]. Furthermore, the extraarticular manifestations that often determine the severity and comorbidity of RA were also closely associated with ACPA positivity [74]. Therefore, although disease onset can follow a similar course, the erosive and destructive nature of ACPA+ RA has resulted in clinicians and scientists considering the diseases as two distinct entities [75]. The main clinical use of these antibodies is however their diagnostic value, now recognised for over 25 years [45–50, 76] but only more recently used as a diagnostic biomarker. Sensitivity (*∼*40%) and specificity (over 95%) of ACPA as diagnostic biomarker are now recognised in early inflammatory arthritis patients with a suspicion of RA [54].

A study using matched serial serum samples (blood donations) from early RA patients with short disease duration highlighted the importance of ACPA in predicting disease severity [50, 77]. The results also showed that radiological damage was more apparent in the groups which had been ACPA+ even before diagnosis was achieved. Radiological progression was also more substantial in this group after 2 years of follow-up. Importantly, these associations were not observed with RF. In contrast, ACPA titres were reduced over the course of disease when patients had a good response to therapy and titres of ACPA at baseline were higher in patients with poorer response. Taking this a step further, van Gaalen and colleagues prospectively studied a cohort of patients at an earlier stage of the disease in order to determine which markers may predict disease progression and persistence [78]. Individuals with an inflammatory arthritis but who did not fulfill the American College of Rheumatology (ACR) classification criteria were recruited. Multivariate analysis confirmed ACPA as an important independent predictor of RA with 93% developing RA within 3 years if ACPA+ at baseline. Given the clinical relevance of ACPA, it is not surprising that the new ACR/European League Against Rheumatism (EULAR) 2010 RA classification criteria have included ACPA titre in order to improve the diagnosis of early RA [79].

Studies which have evaluated ACPA titres while treating RA are emerging with variable observations (recently reviewed in [80]). Conventional antirheumatic drugs (DMARDs, including methotrexate, hydroxychloroquine, minocycline, or sulfasalazine) induce a marginal reduction in ACPA titres (>25%) over the course of treatment in about 50% of patients and a more pronounced decrease (>50%) in less than 30% of patients [81]. Response to TNF blockade was associated with lower baseline titres for ACPA, other clinical parameters being similar [82]. Response was also associated with a sustained reduction in ACPA titres, other studies showing similar *∼*30% reduction of serum ACPA titres after anti-TNF treatment [81–92]. However, several other reports showed little or no effect on ACPA titre [93–97]. Therapeutic B-cell depletion (using Rituximab an anti CD20 antibody depleting naive, memory, and preplasma cells but not plasma cells) has marginal effect on ACPA titres [98–101] or not at all [102]. Significant reductions of ACPA titres were only observed in patients who responded to chemotherapy and higher titers of ACPA were associated with lack of clinical improvement [103].

## 3. Anticarbamylated Protein Antibodies (Anti-CarPA) in RA

The first demonstration of the deleterious effects of protein carbamylation in humans was made in the 1970s [104]. The quantification of carbamylation-derived products (CDPs) remains rarely used in clinical practice [105] and evaluation of antibodies against carbamylated proteins is just emerging. PTM through carbamylation has been implicated in vascular dysfunction in renal disease, atherosclerotic plaque formation [106], and antibiotic resistance [107].

### 3.1. Chemical Reaction

Unlike citrullination which is catalyzed enzymatically, carbamylation (often referred to as homocitrullination) is a chemical modification. It can occur ubiquitously in the presence of the reactive metabolite, cyanate. One of the cyanate sources is the spontaneous degradation of urea, which is constantly and ubiquitously generated in the body and always in equilibrium with cyanate. Therefore, wherever there is urea, there is cyanate and the potential for homocitrullination. However, under normal physiological conditions, concentrations of both are too low for any significant proteins modification.

Theoretically, any protein can be carbamylated* in vivo*. However, the susceptibility of each protein to such modification depends on various parameters, such as the number and accessibility of lysine and arginine amino groups, and the protein lifespan. As carbamylation is nearly irreversible, it is more likely to affect long-lived proteins as they may acquire homocitrulline residues over time [9]. Various CDPs can be formed, among them *α*-carbamyl-amino acids (or *α*-carbamyl-proteins) when *α*-amino groups are involved, and *ε*-carbamyl-lysine, also called homocitrulline, when *ε*-amino groups are involved (Figure 1(b)) [104]. The carbamylation of amine groups leads to a change in the charge of the molecule. Carbamylated derivatives may therefore acquire biological and antigenic properties that are different from those of the noncarbamylated molecules. On the other hand, carbamylation-induced conformational changes in proteins are also associated with partial or complete loss of protein functions [26], inhibition of enzymatic activities particularly relevant in RA such as matrix metalloproteinase-2 [108] or tissue inhibitor of metalloproteinase-2 [109], modification of hormonal activities (i.e., insulin [110], glucagon [111], adrenocorticotropic hormone [112], and erythropoietin [113]), and by affecting proteins such as haemoglobin [114], albumin [115], and collagen [116, 117].

### 3.2. Clinical Relevance

Carbamylated proteins may have a role in inflammation and as such in RA. They can modulate the functions of inflammatory cells, as evidenced by the inhibitory effect of carbamylated-albumin on the polymorphonuclear leukocyte respiratory burst [115, 118]. Carbamylation of low density lipoproteins (LDLs) by myeloperoxidase (MPO) seems to play a pivotal role inatherosclerosis [119–122] as well as in inflammation [106, 122–124]. Carbamylated collagen stimulates the production of active matrix metalloproteinase-9 (MMP-9) by monocytes, thus potentially enhancing extracellular matrix turnover [104, 125]. Therefore it is intriguing that homocitrulline also represents an immune target in RA.

In 2010, the presence of anti-CarPA (also called antihomocitrullinated protein/peptide antibodies; AHPA) was demonstrated [26] in human sera and in an animal model of autoimmune arthritis expanding the set of known autoantibodies related to RA. Reactivity to carbamylated animal protein has been reported but the exact nature of the autoantigens recognised by anti-CarPA remains elusive. Fibrinogen is extensively accessible to homocitrullination and there are substantially more potential amino acid residues available for this type of modification in this molecule compared to citrullination [118]. The generation of antibodies to carbamylated regions of fibrinogen in RA patients was confirmed [118]. The RA specificity of anti-CarPA was suggested (*n* = 84) as these antibodies were not found in patients with other inflammatory rheumatic conditions SLE (*n* = 37, 5% weakly positive results) and psoriatic arthritis (*n* = 37, 3% weak reactivity) or normal healthy individuals (*n* = 27). The fact that some RA patients have reactivity to carbamylated but not citrullinated fibrinogen supports the concept that homocitrullination can generate unique structural antigens on proteins, that is, although cross-reactivity between ACPA and anti-CarPA was recently reported [118]. In another study carbamylated vimentin was used to detect anti-CarPA in RA patients [126]. Carbamylated vimentin was significantly more reactive than carbamylated enolase which suggests that the amino acids surrounding the modification (or even the whole molecule) are contributing to its immunogenicity [126]. The known association between ACPA and MHC class II SE expression [127, 128] was very recently supported for anti-CarPA with data showing that homocitrulline and homocitrullinated peptide could potentially bind to the SE [118].

Anti-CarPA IgG were found in the serum of 45% of RA patients and IgA anti-CarPA in 43% [9]. The presence of anti-CarPA partially overlapped with the presence of ACPA, but most interestingly was also found in 16% of RA ACPA− patients (30% were positive for anti-CarP IgA) [9]. The presence of anti-CarPA was detected in over 30% of such patients when ACPA− therefore offering an alternative biomarker to help the diagnostic of RA [9]. Furthermore, anti-CarPA positivity was related to clinical outcome [9]. Detection of anti-CarPA at disease presentation was predictive of a more destructive disease course (evaluated using Sharp-van der Heijde scores). Importantly, this was verified in both ACPA+ and ACPA− RA, notably offering a novel biomarker for the diagnostic of RA and, furthermore, a clinically useful prognostic biomarker for ACPA− disease.

In individuals with seropositive arthralgia (340 patients positive for rheumatoid factor (IgM-RF) and/or ACPA+), the prevalence of Anti-CarPA was 39% [129]. The presence of anti-CarPA did not correlate with RF. Anti-CarPA were associated with progression towards RA. Furthermore, established association indicated that anti-CarPA positive arthralgia patients were more likely to develop RA and notably within a shorter time frame compared to individual with only RF and/or ACPA positivity. Such increased risk of developing RA was maintained in double positive ACPA/anti-CarPA arthralgia patients even after correction for ACPA. Higher anti-CCP antibody levels were also observed in anti-CarPA positive patients. These observations suggest that alternative seropositivity in RA patients may each represent a different disease entity with its own genetic/environmental contributions [129, 130].

Despite these promising initial findings, further research is needed to clarify anti-CarPA responses and how they could contribute to the clinical management of RA. Additional studies using patients with a suspicion of RA as controls are needed to determine the specificity of anti-CarPA for RA diagnostics. Whether their presence predicts the development of (ACPA−) RA in patients suffering from unclassified joint complaints such as arthralgia or early signs of inflammatory arthritis remains to be established [9, 130]. Links with environmental factors (smoking, alcohol intake, body mass, hormonal status, periodontal disease, etc.) remains to be elucidated. Despite the association with SE, other genetic factors may be relevant. Early aggressive treatment in RA has been shown to prevent future damage [131, 132]. The clinical utility of a prognostic biomarker such as anti-CarPA in the management of ACPA− patients with respect to their risk of developing a more severe disease remains of great interest [9].

## 4. Antioxidized Protein Antibodies in RA

Oxidative stress is a term that is used to describe situations in which an organism's production of oxidants exceeds the capacity to neutralize them. The consequences are damages to cell membranes, lipids, nucleic acids, proteins, and constituents of the extracellular matrix such as proteoglycans and collagens. Several lines of evidence suggest a role for oxidative stress in the pathogenesis of RA [133–139]. Epidemiologic studies have shown an inverse association between dietary intake of antioxidants and RA incidence [140–143], and, reciprocally, an inverse association between antioxidant levels and inflammation [39, 144, 145]. Reactive oxygen species (ROS) are chemically reactive molecules containing oxygen (such as superoxide and peroxides), and a natural byproduct of the normal metabolism of oxygen. ROS are able to oxidize various amino acids, according to their oxidation potential. They have important physiological roles in cell signaling, apoptosis, ion transport systems, wound healing and blood homeostasis, and also the induction of host defense (respiratory burst), genes, and inflammatory responses. They can also be detrimental in situations of stress when their levels dramatically increase to the point of harming cells. This notably occurs when antioxidants normally protecting cells (superoxide dismutases, catalases, peroxidases, peroxiredoxins, and others) are unable to manage the amount of ROS produced [146].

Oxidative modifications by ROS are attractive candidates as instigators of autoimmunity and this might involve a process of “oxidative PTM intolerance” [10], resulting in a primary B-cell response against the posttranslationally modified self-antigen [10]. Oxidative stress-induced antibodies to carbonyl-modified protein have also been found to correlate with severity of chronic obstructive pulmonary disease [147] and SLE [148].

### 4.1. Chemical Reaction

Oxidative stress occurs during inflammation and causes proteins to become damaged by reactive species such as reactive oxygen, nitrogen, and chlorine species. NADPH oxidase is a major source of ROS in arthritic joints. This enzyme reduces O_2_ generating large amounts of superoxide radical anion ^∙^O_2_^−^, which is considered the primary ROS and may be further reduced to H_2_O_2_, which in turn can be converted into highly reactive  ^∙^OH  or react with Cl^−^ to generate HOCl (in a reaction catalyzed by the enzyme myeloperoxidase). iNOS also generates  ^∙^NO  which is converted to ONOO^−^ by reacting with O_2_^•−^ [149, 150]. In addition, under conditions of oxidative stress, species such as peroxynitrite (ONOO^−^) may be generated resulting in nitration of tyrosine residues to form 3-nitrotyrosine (3-NT) (Figure 1(c)) [138, 151, 152]. Indeed, antibodies recognizing 3-NT have been identified in the synovium of RA patients and correlate with disease activity [152].

In addition, these reactive species generate “secondary” reactive species such as lipid peroxidation products. Nonenzymatic oxidation by sugars can react directly or generate reactive products such as glyoxal and methylglyoxal; these reactive carbonyls are capable of undergoing Maillard reactions, first forming a Schiff base with the amine group of amino acids, such as lysine or arginine. This intermediate can then undergo an Amadori rearrangement to form stable advance glycation end product (AGE) such as carboxymethyl arginine or initiate peptide cross-linking to form pentosidine (Figure 1(d)) [153]. The presence of these PTM on protein increases as well as modifies their natural antigenicity and antibodies against the native and modified forms of these proteins are usually noncross-reacting and were detected in RA despite the absence of hyperglycemia [17, 154]. AGEs can have damaging effects on collagens by forming irreversible cross-links between the fibers in the triple helix [155–158].

Another potential reaction is chlorination of aromatic amino acids, in particular tyrosine residues, including 3-chlorotyrosine, within the polypeptide backbone (Figure 1(c)) [159]. Under conditions of oxidative stress, species such as peroxynitrite (ONOO^−^) may be generated resulting in nitration of tyrosine residues to form 3-nitrotyrosine (3-NT) (Figure 1(c)) [138, 151, 152]. Indeed, antibodies recognizing 3-NT have been identified in the synovium of RA patients and correlate with disease activity [152]. Exposure of collagens to peroxynitrite results in nitration of tyrosine residues and formation of posttranslationally modified nitrotyrosine. These compounds are negatively charged and further disrupt the collagen structure. ROS levels are increased in autoimmune diseases such as RA and SLE. The overproduction of ROS may exceed the capacity for radical scavenging by antioxidant enzymes or small inhibitors. Exposure of proteins, nucleic acids, or cell membrane and free lipids to ROS modifies amino acids creating PTM proteins and lipids by initiation of peroxidation. There is no recognized specificity to the protein that can be modified and oxidation depends on steric and stochastic factors; however, enrichment for amino acid motif YXXK in the vicinity of chlorination has been observed [160]. Oxidized proteins identified in RA include collagens I, II, IX, and XI, proteoglycans, and hyaluronan. Increased oxidation of lipids is also a known feature of RA, with the appearance of foam cell-like structures within the rheumatoid synovium [39, 136, 161].

In the context of RA, immunoglobulins themselves can undergo glycation to generate AGE-IgG. Autoantibodies to such modified-IgG were also shown to be specifically associated with RA, whereas the actual formation of AGE-IgG was directly related to the intensity of the inflammatory response but was not specific to RA [162–164]. Similarly, modification of IgG by HOCl or peroxynitrite can induce a T-cell response against IgG HOCl and peroxynitrite in RA [165].

### 4.2. Clinical Relevance

The key ROS present in inflamed joints are superoxide radical (O_2_^∙−^), hydrogen peroxide (H_2_O_2_), hydroxyl radical (^∙^OH), hypochlorous acid (HOCl), nitric oxide (^∙^NO), and peroxynitrite (ONOO^−^), which are involved in acute and chronic inflammation [6, 15]. Such ROS have been identified in synovial fluid of 90% of patients with RA, with a shift in the oxidant/antioxidant balance in favour of lipid peroxidation, which lead to the tissue damage observed in joints [166, 167].

Exposing CII to conditions which simulated those found in an inflamed joint, resulted in chemical modifications of native CII [6]. CII treated with hydroxyl radical (^*∙*^OH-CII), hypochlorous acid (HOCl-CII), and peroxynitrite (ONOO^−^-CII) demonstrated positivity for binding to autoantibody specifically recognizing these various CII-modified forms in sera from 93 of early RA patients in addition to glycation of CII (Gly-CII) carried out with ribose. No cross-reactivity with native CII was observed but reactivity to native CII was seen in <20% of sera [6]. Moreover, no anti-ROS-CII reactivity was detected in other inflammatory arthritis conditions (including psoriatic arthritis, SLE, ankylosing spondylitis, palindromic arthritis, scleroderma, Behçet's disease, primary Sjögren's syndrome, fibromyalgia, tendonitis and reactive arthritis [6].

In 2005 we showed that CII post-translationally modified by ROS (ROS-CII), present in the inflamed joints, is an auto-antigen in RA [6]. In addition, cartilage damage as a result of collagen oxidation by glycation and formation of AGE-CII are evident despite the absence of hyperglycemia [168].

We have recently measured auto-reactivity to ROS-CII in synovial fluid (SF) and serum samples taken from various phases of RA [38] and demonstrated that anti ROS-CII reactivity is not related to markers of inflammation such as CRP and ACPA and has potential to serve as biomarker for several purposes. We observed high anti-ROS-CII reactivity in DMARD naïve early RA regardless of whether patients were ACPA+ or ACPA− and with no correlation with DAS28. The sensitivity and specificity of the binding of autoantibodies to ROS-CII in early RA compared with healthy controls (HC) was 92% and 98%, respectively. ROS-CII reactivity was lower in RA patients having received their first DMARDs treatment and achieving a good response. We also showed that anti-ROS-CII reactivity considerably vary over time in a mixed cohort of RA patients with established disease on several type of treatment [38]. This was in contrast to levels of ACPA which did not. We could not directly associate these changes with DAS28, however, patients in this cohort all had (very) active disease and it was impossible to fully ascertain longitudinal variation between active/remitting diseases.

Further pilot data showed that in a small cohort of ACPA+ arthralgia patients with no synovitis, only those within a few weeks (*∼*12) of developing clinical evidence of synovitis were positive for anti-ROS-CII reactivity while those who developed symptoms after a much longer delay were negative. Interestingly, in a study conducted in type 1 diabetes, a condition associated with RA [169], anti-ROS-CII reactivity was restricted to SE-containing DRB1∗04 alleles (OR 3.62; 95% CI 1.12–11.74), known to confer the greatest risk for developing RA. Further work needs to establish whether patients with inflammatory synovitis but not yet RA (i.e., undifferentiated arthritis) would be positive however, 93% of early RA were, which altogether, strongly suggest a direct association with the development of synovitis, hence offer a measurable biomarker of disease development alongside the RA continuum [38].

Anti-TNF treatment showed reduction in oxidative stress, and these correlated with an improvement in disease activity [170–173]. However studies evaluating changes in anti-ROS autoantibody levels after RA anti-TNF treatment are still missing. Our own data however suggest variation [38] which will need to be confirmed before any biomarker value can be confirmed.

In addition to CII, studies of RA synovial fluid and tissue have demonstrated oxidative damage to hyaluronic acid [174], lipid peroxidation products [175, 176], oxidized low-density lipoproteins (ox-LDL) [136], and increased carbonyl groups reflective of oxidation of other proteins [136, 162, 177–179]. Evidence of oxidative damage to cartilage, extracellular collagen, and intracellular DNA has also been demonstrated. Protein chlorination occurs in RA at the disease site (i.e., synovial fluid and tissue) [159, 180] and it was proposed that this could be the link between arthritic inflammatory reactions and the initiation of autoimmune antibody responses. The risk associated with ox-LDL in RA is mostly related to cardiovascular risk hence not specific to RA. Ox-LDL are strong autoantigens, essential to the development and progression of the plaque in atherosclerosis as LDL molecules only become immunogenic due to the oxidative modification during early atherogenesis [181, 182]. Anti-oxLDL antibodies are extensively prevalent in patients with autoimmune diseases, including RA [183], SLE [184, 185], and antiphospholipid syndrome (APS) [186, 187], diabetes mellitus [188, 189], uremia [190]. Anti-ox-LDL antibodies bind ox-LDL and generate immune complexes. Circulating immune complexes are not in themselves harmful. They cause damage only if they are deposited in tissues (notably in the endothelium), resulting in inflammation [191, 192]. T-cells, primarily CD4^+^ cells, have been found associated with these immune complex depositions [193–196]. Cardiolipin is also the target of oxidation (ox-CL). Anti-ox-CL antibodies are frequent in APS patients [197, 198] due to formation of neoepitope on cardiolipid, possibly with cross-reactivity with anti-oxLDL antibodies notably in patients with SLE [197, 199, 200].

Finally, autoantibodies targeting AGE-modified IgG are also present in serum of RA patients [154, 201]. Autoantibodies against AGE-IgG might be helpful in monitoring progress in the RA disease continuum and in combination with other clinical features of the RA might be a useful diagnostic tool [201].

## 5. Animal Model Testing of PTM-Targets and Antibody to PTM-Proteins

An important discovery in the association between anti PTM-protein and RA was the demonstration that these antibodies and their targets are both arthritogenic in animal models. The citrullinated forms of collagen II appeared more arthritogenic in rats than native collagen II [202, 203]. Within the human synovium, the immune reaction between citrullinated fibrin and ACPA results in the activation of effector mechanisms. Immune-complex containing ACPA and CII citrullinated peptide can activate blood macrophages via FcR resulting in the production of TNF-alpha in mice [204, 205]. A similar response by synovial macrophage would promote local inflammation which in turn will favour plasma extravasation and fibrinogen polymerisation. These deposits then could get citrullinated by locally expressed PAD and therefore become new target for ACPA closing the circle for self-perpetuation. PAD 2 and 4 are expressed in the RA synovium (and in other inflamed tissues) but importantly in correlation with the intensity of inflammation [65]. The arthritogenicity of chlorinated-CII versus native CII (Cl-CII) was also demonstrated in a rat strain [159, 206, 207]. This might be caused by an increased immunogenicity of Cl-CII, resulting in a stronger antibody-inducing capacity. Hydroxyl radical modification of collagen type II (OH-CII) also increases its arthritogenicity and immunogenicity and resulted in an early and more severe arthritis compared to native CII [208].

Anti-CarPA are now extensively studied to clarify whether they are directly involved in the pathogenesis of RA. carLDL induce an IgG response in LDL-R^−/−^ mice and autoantibodies also bind to humans plasma proteins [209]. The immunogenicity and an arthritogenic role of the antihomocitrulline immune responses were confirmed using animal model of arthritis. Immunization of several mouse lines (NMRI, BALB/c, and C57bl/6) with carbamylated-peptides leaded to a Tcell dependent activation of B-cell and the production of autoantibody [26]. Direct intra-articular injection of the carbamylated-peptides in these mice induced a severe erosive arthritis [26]. This study was also the first to report the presence of anti-CarPA in RA patients, both in the joints and circulation, and importantly in relation to erosions. Rabbits immunized with carbamylated-proteins resulted in high-affinity antibodies to homocitrulline-containing collagen telopeptides and to less strong anticitrulline-containing telopeptides and mutated citrullinated vimentin [27].

The exact pathogenic potential of anti-CarPA therefore appears to be similar to that of ACPA [210]. The possibility of cross-reactivity between these two antibody types demands further investigation into the identification of true targets in RA. If antibody responses to citrulline and homocitrulline are indeed arthritogenic, important questions remain: which antibodies are pathogenic? Is it the specificity of the target antigen, the quantity, and diversity of the response, and/or merely the binding affinity to available targets in the arthritic joint, which are important in determining arthritogenicity and clinical disease progression [211]?

## 6. Autoantibodies and B-Cell Development in RA

To date the overall development of the anti-PTM-protein antibody producing B-cell clones remains poorly understood. ACPA of the IgG are the immunoglobulin isotype specifically associated with RA [57]. This suggests that an immune reaction leading to the development of IgG ACPA is taking place at some point before the onset of RA. ACPA of the IgG subclasses 1 to 4, are detected; however a major bias is observed towards an IgG1 (86% alone) and IgG4 but with a very limited involvement of IgG2 and 3 [57]. Such bias correlates closely with an imbalance toward Th1 polarisation which is well described in RA.

The presence of B-cell reactivity to Cl-CII in RA patients was established [6, 38]. Spontaneous production of ACPA could only be obtained from B-cells isolated from the synovial fluid and bone marrow of IgG ACPA+ RA patients. The presence of IgG ACPA up to 15 years before symptoms has also been reported [44, 212]. A cross-sectional study also reported that titres of IgG ACPA appeared higher shortly before the onset of RA suggesting reactivation of the producing B-cells [44]. Finally, the strongest argument in favour of this immune reaction is the T-cell response to citrullinated peptide observed in RA patients but not in healthy controls [213, 214].

The hypothesis that each stage of the disease represents an evolution in ACPA specific B-cell maturation is therefore attractive. At this stage, however, it has not yet been either demonstrated or nullified. ACPA have been shown to be present at detectable levels years before the first manifestation of RA with high risk for these individuals to develop RA within 5 years [22, 124]. In the preclinical phase (ACPA positivity but no disease symptoms), ACPA-IgG circulate (sometimes for many years) suggesting that, at least, isotype-switched ACPA-specific B-cells are present. During this disease initiation phase, cross-sectional analysis also showed that ACPA titres are higher just before onset of symptoms [215–217].

*In vivo*, differences in ACPA levels [73], fine specificity or epitope spreading [218, 219], avidity [220–222], isotype usage [223], and glycosylation [224] may be associated with differences in the potential to activate effector mechanisms, thereby influencing their biological potency [220]. Epitope spreading is often a hallmark of progressive B-cell responses and was described for ACPA and was associated with an increase and/or shift in antigen recognition during the course of an autoimmune response [218, 225, 226]. Fine mapping analysis of preclinical sera compared to early and established RA showed subtle difference in either the identity or the numbers of epitope detected between the different phases of the disease [43, 215, 216, 227, 228]. Our own unpublished data using the same platform showed a particular epitope detected exclusively in synovial fluid which may represent a unique specificity with local retention of the ACPA (as not detected in sera) suggesting local B-cell reactivity. Despite the association between the presence of anti-CarPA and the broadening of ACPA's fine specificities, anti-CarPA are generated independently of ACPA and, to date, are largely noncross-reactive although the panel of currently available carbamylated antigens remains limited. The effect of anti-CarPA in arthralgia patients is notably independent of the effect of ACPA (after correction) [129]. It will be of great interest to expand the investigation for anti-CarPA and anti-ROS specificities, particularly among ACPA− patients and determine whether these antibodies could have pathological effects in RA patients [229].

A few studies already have shown that circulating ACPA-IgG differ in avidity but still relatively little is known about avidity maturation of ACPA before and during the RA continuum [220]. Lower ACPA avidity was reported in ACPA+ asymptomatic individuals compared to avidity in ACPA+ patients with joint symptoms (arthralgia), which was similar to avidity observed in established RA patients [221, 222]. Following immunoablative therapy, ACPA-IgG of low avidity developed again which suggested a newly generated autoimmune response [103]. However, the development of high avidity ACPA-IgG remains speculative and their presence may be only characteristic for specific RA patients, refractory, or less responsive to immunosuppressive treatment [103].

As mentioned previously, all immunoglobulin isotypes (IgM, IgA, and IgE ACPA) contribute to overall ACPA activity in RA serum [223, 230, 231]. Although autoantibodies of IgG isotype are generally the most relevant, other studies have shown that IgA were also specific for RA [231, 232]. IgG are associated with radiographic progression in RA [73, 77], but patients positive for IgA-ACPA with recent onset RA were reported to suffer a more severe disease course over the first three years [233] and the higher the number of different isotypes, the greater long-term radiographic joint damage at 5-year follow-up [234]. This data suggests that the development of the anti-CCP isotype repertoire occurs early in the course of arthritis [217, 235].

Glycosylation of the Fc-part of antibodies affects their function with either a pro- or an anti-inflammatory outcome functionality [236]. The glycosylation profile of ACPA in RA is characterised by a low content of galactose (hypoglycosylation) and sialic acid residues [224]. Hypoglycosylation of ACPA was more pronounced than that of total IgG1, resulting in a proinflammatory Fc-glycosylation pattern of ACPA that could be one mechanism driving inflammation in RA [224, 237]. Fc-glycosylation of ACPA showed significant differences between SF and serum and, in contrast to ACPA in serum, ACPA isolated from SF were found to be highly agalactosylated [224]. IgG glycosylation showed association with RA activity [238]; however, this pattern was not useful to predict clinical response to MTX and anti-TNF treatment in RA [239]. Finally, the specific ACPA-Fc hypoglycosylation was detected already 6 months prior to RA onset [237].

TNF-alpha is an important factor in GCR. If disease initiation was to coincide with a time when B-cells are undergoing early TNF/GCR dependent maturation phases, TNF-blockade in early disease should result in definite ACPA titre reduction. Studies of the effect of TNF blockade in early disease are still lacking and are in progress. In established disease TNF-blockade is clinically efficient but may not be able to interfere with the course of B-cell differentiation anymore; hence studies analysis ACPA titres over the course of anti-TNF therapy in established disease showed variable results. In long lasting RA, B-cell ablation does not result in major ACPA titres reduction in contrast to total IgM titres (but not IgA and IgG) [98–102]. Plasma cells not being directly depleted by the therapy due to the fact that they do not express CD20, suggesting that, in established RA, ACPA-LL-PC are present. The small reduction in ACPA titres reported after B-cell depleting therapy (< than 20–30%) nevertheless suggests that a small pool of ACPA producing cells (memory and SL-PC) are affected by the therapy [240] notably as SL-PC were evidenced in the synovium of RA patients and were shown to secrete autoantibodies including ACPA [241, 242].

The direct analysis of ACPA producing B-cells has proven difficult. The classic molecular tools used to label antigen-specific B-cells have not been very successful to date in isolating ACPA-B-cells (MHC-tetramers, biotinylated-peptide specific for ACPA BCR). ACPA-specific T-cell clones were detected in established disease [213]. However, it remains to be determined whether they play a role in anticitrullination response in RA and most importantly when. The further elucidation of the B-cell maturation path will require serial samples from preclinical stages, then early and fully established disease and the examination of somatic hypermutation and affinity maturation.

Data generated to date therefore establish the presence of an immune reaction resulting in the secretion of ACPA. Yet, the primary stimulus leading to such production remains unknown. An environmental association between the presence of ACPA and smoking has been established [219, 243], and smoking is the most recognised environmental factor reproducibly associated with RA. Recently, silica exposure has also been linked to RA [244–246] and other immunologically mediated diseases [247]. A study looking at the link between genetics and environmental factors has shown that the presence of ACPA was associated with the shared epitope HLA-DRB1 in a dose dependent manner but that smoking was only important in patients positive for ACPA secretion [75, 248, 249]. This observation may suggest that physiological processes associated with smoking have a role in the initial generation of ACPA. A model has been proposed in which smoking (and other agents) triggers the production of IgG ACPA [250]. A second event leads to the citrullination of synovial proteins which would direct ACPA immunity towards the joints [218]. The disease would then be initiated, and, if uncontrolled, become chronic. The role of ACPA in the self-maintenance of RA, once it is established, is a more easily understandable model; however, the exact nature of the citrullinated protein target of ACPA remains elusive.

Therapeutic B-cell depletion results in disease improvement (by 6 to 10 weeks) but not in ACPA serum titres reduction. Synovial depletion of B-cells however is delayed (26 to 30 weeks) probably accounting for the time necessary to eliminate short-live plasma cells from the tissue [98]. Therefore some of the benefit of the therapy must be related directly to the removal of B-cell (not plasma cells) from the tissue. There has been speculation that synovial B-cells in RA may have some unusual lack of responsiveness abolishing their proliferative capability (anergic B-cells) leaving intact their antibody production [251]. Two-way interaction between B-cells and T-cells may be a great relevance here: B-cells provide signals to T-cells through antigen presentation and T-cells provide “help” to B-cells through the delivery of cytokines and cell mediated stimulation, creating a self-sustained feedback loop. Whether B-cells stimulate T-cells to stimulate B-cells, vice versa or, more simply, which cell makes the initial mistake and trigger autoimmunity has been a point of debate for years [252]. In favor of B-cell, an argument has been put forward for CD4^+^ T-cell activation being dependent on B-cells in the synovium, in the context of a GCR and in a HLA-DRBI restricted manner where the antigen is harbored by the B-cells [253]. Breaking this loop should, in itself, restore self-tolerance. Therefore, the removal of ACPA secreting B-cell may be more relevant to reestablishing self-tolerance in RA as it may remove ACPA themselves but also the source of activation for the two-way interaction between B- and T-cells.

## 7. Conclusion

The findings presented in this review support the hypothesis that PTM of self-antigens, in RA and in inflammation in general, are a cause of the formation of neoepitopes giving rise to autoantibodies. Whether the breakdown of tolerance occurs because antibodies against modified self-protein are promiscuous and bind both the modified and unmodified self-antigen or whether they are truly specific for modified proteins is unclear. Nevertheless, these processes contribute to the vicious circle of chronicity by providing novel immune reactivity, resulting in further stimulation of the immune response against self-antigens. With the advancement of research methodology it should be expected that novel specificities of autoantibodies against PTM proteins could be discovered in patients with autoimmune diseases. Many of these autoantibodies could have significant biomarker potential. Clearly, animal models suggested therapeutic advantages in preventing the generation or binding of potentially pathological autoantibodies to the extracellular matrix collagen and collagen-like structures. B-cell responses to native CII have long been known in RA but, as PTM-CII reactivity induces or worsens experimental arthritis, it is possible that blocking PTM could ameliorate arthritis [7, 206]. As such, antioxidants and inhibitors of oxidative enzymes have already been shown to ameliorate arthritis in animal models [254, 255]. The translation of antioxidant therapies to human clinical studies has produced disappointing results, but targeted approaches using novel inhibitors of oxidative enzymes offer new hope for the treatment of RA.
